# Limitations of current spatial ability testing for military aviators

**DOI:** 10.1080/08995605.2021.1965786

**Published:** 2021-10-28

**Authors:** Joseph T. Coyne, Sabrina Dollinger, Noelle Brown, Cyrus Foroughi, Ciara Sibley, Henry Phillips

**Affiliations:** a Information Technology Division, Naval Research Laboratory, Washington, DC, USA; b Operational Psychology, Naval Aerospace Medical Institute, Pensacola, Florida, USA

**Keywords:** Spatial ability, selection, classification, aviation, ASTB

## Abstract

Spatial ability has long been considered an important attribute when identifying military aviators. This paper examines the Direction Orientation Task (DOT), which is currently used by the US military to assess spatial ability in aviation applicants. Several limitations of the test, such as a limited number of trials and the availability of practice trials online, make it subject to potential ceiling effects. The paper presents historical data of all Naval Aviator applicants over a six-year time period and revealed that 22% of applicants answered 90% or more of the questions correctly. Furthermore, test performance has significantly increased in the years since the test was first administered and there is evidence that DOT is no longer contributing incremental validity. A follow-up empirical study looked at DOT performance and strategy in a group of military student aviators and student air traffic controllers. The results of the empirical investigation reveal that the use of an analytic strategy was associated with higher performance on the DOT, whereas the use of a spatial strategy was not associated with performance. Taken together, the improved performance data over time and the data on strategy use suggest the test’s ability to measure spatial ability may be diminishing, ultimately reducing its construct and incremental validity. This is problematic and should be addressed, since the DOT is the only measure of spatial ability used by the Navy to assess aviation applicants.

**What is the public significance of this article?—**The data presented here suggests that the Direction Orientation Task (DOT), the only test of spatial ability used to select pilots for the Navy, Marine Corps, and Coast Guard, should be replaced. Applicant scores on the DOT continue to rise each year suggesting that the approach or strategy applicants are using for the test are changing.

As the world’s largest employer, the US military faces a number of unique personnel selection challenges. One specific problem is identifying future military aviators from an applicant pool that has little or no aviation-related experience (Hunter & Burke, 1994). This is very different from how the commercial airline industry selects pilots since their main criterion for evaluating applicants is number of flight hours, a direct measure of aviation experience. The training attrition rates for military aviation were around 25% in the 1980s (Hunter, 1989) and more recent data from the US Navy show a modest reduction with attrition for pilots being about 17% and flight officers 23% (Arnold & Phillips, 2008). Given the costs in both time and money required to train aviators, further improvements in identifying which applicants will be successful in training can provide substantial savings to the US military. Cognitive ability assessments have been core measurements included in military aviation selection exams. This paper focuses specifically on one cognitive ability construct, spatial ability, and how the military currently assesses this ability in its aviation applicants.

A large body of scientific evidence exists which demonstrates that spatial abilities are good predictors of performance, particularly in science, technology, engineering, and mathematics (STEM) fields (Lubinski, 2010). Longitudinal research studies have found that individuals who obtain degrees in these fields have high mathematical and spatial abilities relative to their verbal abilities. This suggests that the assessment of spatial ability, an ability less reliant on acquired knowledge, provides insight into individual differences beyond standard measures of crystallized intelligence. Despite the importance of spatial abilities, they are often not used by schools and companies to select individuals for STEM degrees or careers (Lubinski, 2010).

Similar to school selection tests, military selection batteries heavily rely upon tests that are dependent on both verbal ability and other learned skills. These types of crystallized intelligence tests often result in subgroup differences based on race and gender. The US military does not solely rely upon measures of crystallized intelligence and has long understood the importance of spatial ability, assessing it in aviation applicants since World War II (Guilford, 1947). Spatial ability is generally considered to be “not acquired by formal instruction” (National Research Council, 2015) and is less prone to subgroup differences. For example, Held, Carretta, and Rumsey (2014) found that the inclusion of a spatial ability test within the Armed Services Vocational Aptitude Battery (ASVAB) helped increase the number of qualified applicants from minority groups by reducing subgroup differences without reducing predictive validity. Despite this history of including spatial ability measures, our understanding of what spatial ability is and how it is best assessed is still evolving (National Research Council, 2015).

Lohman’s (1996) definition of spatial ability, “the ability to generate, retain, retrieve, and transform well-structured visual images whose properties include location, size, distance, direction, separation, connection, shape, pattern, and movement” is one of the most widely used. Researchers are in general agreement that spatial ability is a multi-faceted construct (e.g., Carroll, 1993; Fleishman, Costanza, & Marshall-Mies, 1999; Lohman, 1996). Although it is beyond the scope of this paper to discuss in detail the different factors identified by different researchers, Hegarty and Waller’s (2005) chapter provides a good summary. Two factors that consistently appear are spatial orientation: the ability to understand the arrangement of objects in space, and spatial visualization: the ability to mentally manipulate visual objects.

Barron and Rose (2013) note that although there have not been many criterion-related validation studies with respect to the different subcomponents of spatial ability and pilot performance, it is expected that pilots need to possess such abilities. They also note that spatial orientation has the most face validity with respect to piloting. Navigating routes and understanding one’s position within their route, as well as understanding the orientation of objects viewed through a screen or window are important skills for aviators. Other components of spatial ability, such as flexibility of closure: the ability to identify patterns in clustered visual environments, may be important in aviation tasks such as recognizing objects that are partially obscured by weather (Barron & Rose, 2013).

Recently published research suggests that spatial ability may not provide much incremental validity beyond traditional academic tests in predicting military aviation training performance (Johnson, Barron, Rose, & Carretta, 2017). However, the authors did find that measures of perceptual speed loaded on the same factor as spatial ability, and that tests of perceptual speed did add incremental validity beyond other selection measures. That analysis was conducted using historical data from the Air Force Officer Qualifying Test (AFOQT) from the 1990s and may not be reflective of the current timed computer-based tests of spatial ability. Research by Barron and Rose (2013) used a more recent sample, which included the latest spatial ability test, the Direction Orientation Task (DOT), and found that it added incremental validity beyond other measures of spatial ability and perceptual speed when predicting training success of officers who completed an Air Force undergraduate pilot training program. The DOT is a component of the Air Force’s computerized Test of Basic Aviation Skills (TBAS), as well as the Navy and Marine Corps’ Aviation Selection Test Battery (ASTB), both of which are used for selection of military aviators. The DOT is the focus of this paper since it is used across the services and is the only test of spatial ability used by the Navy and Marine Corps. Despite strong correlations between spatial ability and fluid intelligence, spatial ability does capture important variance that general intelligence measures do not. For example, De Kock and Schlechter (2009) found that while measures of fluid intelligence were correlated with ground school performance for pilots, measures of spatial ability were more strongly correlated with flight performance.

The DOT (see Figure 1) is designed to assess an individual’s ability to determine the physical orientation of an object that appears in an unmanned aerial vehicle’s (UAV) forward facing camera, while the UAV is flying a fixed heading. The task shows two images: one with a God’s eye map pointed at the ground, conveying the UAV’s heading, and the other representing the camera view from the UAV. The object individuals are instructed to identify is one of four parking lots (North, East, South, and West), a task which requires understanding how the vehicle’s direction of travel influences how the images appear relative to the vehicle. Understanding the orientation of what a pilot sees out of his/her window is believed to be an important skill for aviators, and early evaluators of the DOT stated “the relevance of this test should be clearly apparent” (Olde & Walker, 2006). According to Olde and Walker (2006) the test is designed to be a measure of the spatial orientation component of spatial ability as well as processing speed, since response time is measured and participants are instructed to respond as quickly as possible.

**Figure 1.**
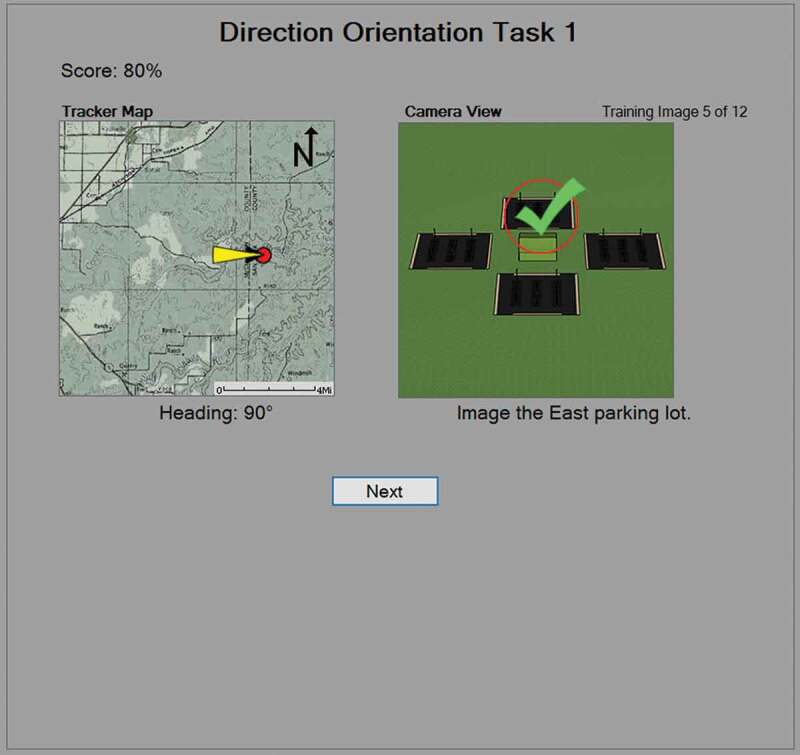
Sample problem from the direction orientation task.

Barron and Rose (2013) looked at how well scores on the DOT, a visualization task (Rotated Blocks), a closure flexibility task (Hidden Figures), and a perceptual speed task (Table Reading) predicted performance for 1,440 USAF officers who completed the Specialized Undergraduate Pilot Training (SUPT), a 22 week course which requires flying the T-6A single engine aircraft. SUPT performance was measured across a series of flights where instructors used Likert scores ranging from 1 to 5 to grade the trainee’s abilities to perform different maneuvers. Means of these maneuver grades are consolidated for each flight, and then again across flights, to yield a single mean ranging from 1 to 5 representing performance at the completion of each major training stage. Barron and Rose (2013) found that all the spatial ability measures were significantly correlated with performance during SUPT. The DOT had the strongest correlation with SUPT daily performance (.149 uncorrected correlation) and accounted for the largest portion of variance (38%) when compared to the other three spatial ability measures. Additionally, in the original validation studies for the different cognitive tests within the TBAS (Carretta, 2005), the DOT was one of the tests that predicted Air Force pilot training success and added incremental validity.

Despite the DOT adding incremental validity in predicting pilot training performance (Barron & Rose, 2013; Carretta, 2005), the test has several issues that have led researchers to begin investigating alternative versions of the DOT (Coyne et al., 2020; Keiser et al., 2019). The main limitation of the current version of the DOT is that there is a ceiling effect (Keiser et al., 2019). However, this has only been shown in data collected in small research samples and not in a larger applicant dataset. There are several reasons why a ceiling effect may be present. Many research studies investigating DOT use restricted samples (i.e., Naval Aviators and Army cadets) who likely have greater levels of spatial ability than the applicant population. Another explanation for the ceiling effect is that the DOT only has 48 possible trials, and with only 4 response options for each trial, a 25% chance of guessing the correct answer to any trial. Additionally, applicants seeking a career in aviation can easily find sample questions to the DOT and other components of the ASTB and TBAS online. Given the small number of trials and the fact each applicant will see every trial, there is a possibility that applicants have memorized all the correct answers (Keiser et al., 2019). Another reason may be the large practice effects associated with the DOT, where students typically improve with subsequent testing (Momen, 2009). If students take practice tests online or study the DOT with flashcards, then those students will likely have higher scores than participants who do not practice the DOT. This could reduce the predictive validity of the test as well as lead to adverse impact since students with the means to practice (finding them online or paying a practice company) will likely perform better than those without the same opportunities.

Another possible explanation of the ceiling effect is that test takers actually apply non-spatial strategies to solve DOT items. Researchers have been looking at ways of modifying the DOT to make it more difficult and possibly reduce the ceiling effect (Coyne et al., 2020; Keiser et al., 2019). The first modification, referred to as the DOT 2, was a change in the imagery on the camera view portion of the display to a ship that was in one of 12 possible orientations, which increased the number of problem combinations from 48 to 144. Keiser et al. (2019) found a group of student Naval Aviators and Flight Officers had a reduction in the average accuracy rate and a decrease in the average response time on the DOT 2 when compared to the original DOT. However, DOT 2 trials can still be solved using a simple math formula: the DOT 2 ship’s actual orientation is equal to the sum of the UAV’s heading and the ship’s apparent direction when that number is less than 360, or the sum minus 360 when it is greater. A subsequent study (Coyne et al., 2020) asked a group of Army cadets to report the percent of time they used this math strategy versus a spatial strategy when solving the DOT 2. They found that the math strategy was reported to be used two times as often as a spatial strategy and that the use of the math strategy was highly correlated (*r = *.74) with accuracy on the task, whereas the use of a spatial strategy had a strong negative correlation (*r = −.6*5) with performance.

Although the DOT and the DOT 2 are somewhat different, a similar mathematical approach can still be used to correctly respond to both tests. However, within the original DOT, another strategy can be applied. If the vehicle is flying in a cardinal direction such as in the sample trial in Figure 1, the parking lot at the top of the map is the UAV’s direction. The parking lot to the right is the UAV’s heading plus 90, and the one to the left is the UAV’s heading minus 90. If the UAV is not flying a cardinal direction such as 240 degrees, the two cardinal directions closest to this heading (i.e., 180 degrees and 270 degrees) will appear at the top of the screen. The parking lot to the top right of the screen would be the cardinal direction with a value greater than the UAV heading (270 degrees), and the one to the top left would be cardinal direction with a value smaller than the UAV heading (180 degrees). Knowing which parking lot(s) is at the top the participant can then apply a counting or addition/subtraction strategy to identify the correct parking lot.

The use of different strategies to solve spatial ability tests is actually fairly common. In fact, Hegatry’s (2010) chapter on spatial intelligence discusses the importance of strategy and being able to flexibly use different strategies to solve spatial tasks as one of the two components of spatial intelligence. However, a group of students applying an analytic strategy to a paper-folding task is different from the issue being discussed here. The DOT is part of a high stakes selection test, and therefore motivated individuals know about the DOT in advance of taking the test and likely practice it. Part of the explanation for the practice effects associated with the DOT could result from examinees developing and using alternative non-spatial strategies found to be related to increased DOT performance (Coyne et al., 2020; Keiser et al., 2019).

The issue of strategy is particularly important for the US Navy and Marine Corps, since unlike the Air Force, they do not use any other measures of spatial abilities for assessing applicants. If the DOT is measuring different constructs depending on the strategy used by the examinee, it may be impacting the predictive validity of the ASTB. Specifically, math aptitude is already being measured by other ASTB subtests, and if applicants are using a mathematical rather than a spatial strategy, the DOT is less likely to add incremental validity beyond those subtests for the prediction of training performance. To date, however, there have not been any studies evaluating different strategies and their relationship to performance on the version of the DOT used in the ASTB and TBAS.

There were two goals of this research. The first was to examine performance on the DOT from recent military applicants. It was expected that the DOT’s ceiling effect, detected in smaller samples of Naval flight students (e.g. Keiser et al., 2019), would also be present in the applicant pool. The second objective was to determine whether strategy use varied across participants and if so, whether strategy use was related to performance. Although alternate versions of the DOT have shown that individuals who use non-spatial strategies perform better (e.g., Coyne et al., 2020), these results have not been demonstrated in the original test version of the DOT. Ultimately, these results will help to inform the US military whether it should consider a new test of spatial ability for the selection of military aviation training candidates.

## Experiment 1

In this experiment, we examined archival data for the DOT from the latest version of the ASTB implemented in 2013, and training data from the primary phase of training for student Naval Aviators.

### Method

#### Participants

Archival DOT data for all US Navy, Marine Corps, and Coast Guard applicants who took the ASTB from December 2013 through September 2020 were examined. This data set includes every applicant during this time period including those who were selected to be aviators and those who were not selected. The Navy allows applicants to take the exam multiple times; however, due to known practice effects within the DOT (e.g., Momen, 2009), only an individual’s first test attempt was included in these analyses. In addition, the year of the applicant’s test attempt was treated as an independent variable. The total number of applicants within this time period was 37,785, and of this population 18% were female.

#### Procedure

The DOT outcome variables for each applicant are the total number of correct responses to the 48 DOT trials, the individual’s median response time across the 48 trials, and the DOT Factor which is a weighted combination of accuracy and response time data. The DOT Factor is the value used by the Navy for selecting aviators, and as such the formula for its computation is not publicly releasable.

Of the applicants whose ASTB data were analyzed as well as an additional 1,511 student aviators who completed an earlier version of the ASTB as well as the performance-based measurement (PBM) portion of the current version (as part of its validation), we were able to obtain Primary Flight Training data for 4,826 student aviators (4,363 of whom completed the primary phase of training). Primary is the second phase of flight training, which follows Aviation Pre-flight Indoctrination (API), an academically intense 5-week didactic program. Two training performance metrics were analyzed: student completion status, a dichotomous variable (i.e. attrite/complete), and Navy Standard Score (NSS) which was only calculated for those who completed training. NSS represents a normed summary of all flight grades, scaled as a T-score, centered at 50 with a standard deviation of 10 and range boundaries of 20 to 80.

### Results

#### DOT applicant data

**Figure 2.**
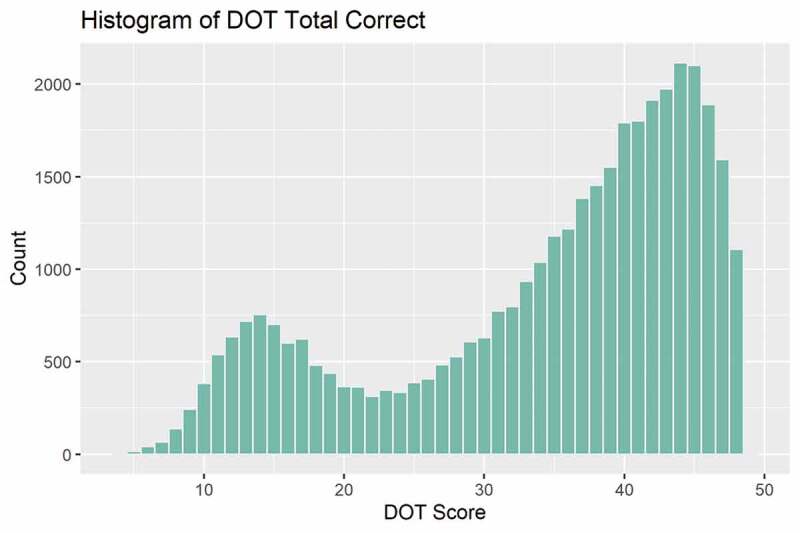
Histogram of DOT total correct for all US Navy and Marine Corps aviation applicants from December 2013-September 2020.

The average number of correctly answered DOT trials was 33.22 (*SD *= 11.22). Figure 2 depicts a histogram of the DOT total correct data for all the applicants. An Anderson-Darling normality test was performed and indicated that the data are not normally distributed, A = 1474.9, *p* < .001. There is a large ceiling effect with 12% of the participants making only 2 mistakes or fewer on the test (greater than 95% accuracy). The median response time for the DOT Trials was also analyzed. The average median response time was 4.06 s (*SD* = 1.98 s). An Anderson-Darling normality test was performed and indicated that the data are not normally distributed, A = 1380.2, *p* < .001.

**Figure 3.**
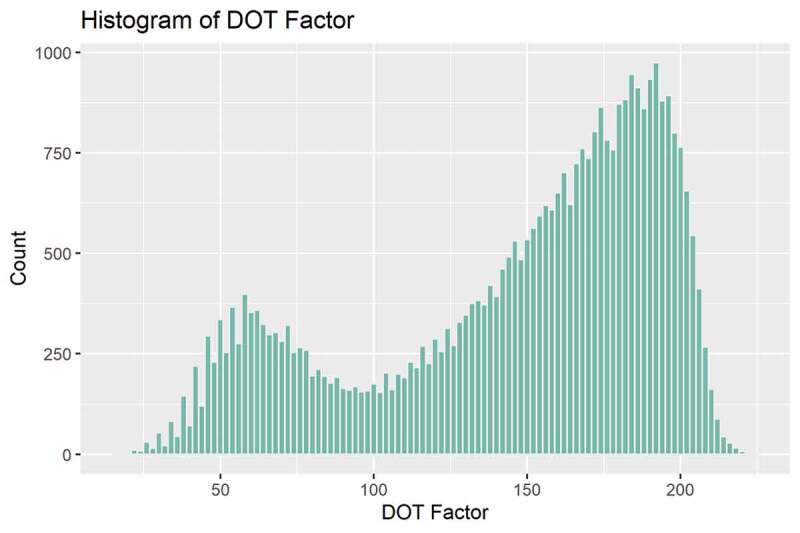
Histogram of DOT Factor (combination of accuracy and response time) for all US Navy and Marine Corps aviation applicants from December 2013-September 2020.

The DOT Factor, the combination of speed and accuracy used to select aviators was also analyzed. Ninety percent of participants scored between 53 and 202 and the mean factor score was 144.7 (*SD* = 48.95). An Anderson-Darling normality test was performed and indicated that the DOT Factor data are not normally distributed, A = 1298.2, *p* < .001. Figure 3 depicts the histogram of DOT Factor data for all the applicants.

Three one-way analyses of variance (ANOVAs) were performed looking at the effect of the year the test was taken on average DOT accuracy, response time, and Factor score. 2013 was excluded in the analysis since there was only ½ a month’s data (155 applicants) since this version of the ASTB began operational use in December 2013. The mean score for 2013 was smaller than every year except 2014; however, there was more variability given the smaller sample. The ANOVA revealed a significant effect of year on test accuracy (*F* (6, 37,623) *= *69.47, *p* < .001). Post hoc analysis using Tukey’s HSD revealed 2014 was significantly lower than every year except 2015, no differences from 2015 to 2017, and then DOT score significantly increased each year from 2018 to 2020. An ANOVA on DOT Factor was also significant (*F* (6, 37,623) *= *70.98, *p* < .001). The post hoc analysis for DOT Factor showed the same pattern of increases as DOT Accuracy. The ANOVA revealed a significant effect of year on response time data (*F* (6, 37,623) *= *2.882, *p* = .008). However, the post hoc analysis only revealed a significant decrease in median response time between 2015 (4.09 s) and 2017 (3.95 s) and between 2015 and 2019 (3.97 s). There were no differences between any of the other years.

#### DOT, performance-based measures, and primary school performance

The DOT is one of several tests included in the PBM portion of the ASTB. The other tests include a dichotic listening task (DLT) in which different strings of numbers and letters are presented simultaneously to each ear on a single trial, and participants are prompted to respond to input presented to a target ear only, by selecting different control inputs based on whether each number presented to the target ear within a string is odd or even. An Airplane Tracking Task (ATT) measures the ability of the applicant to track a moving object in two dimensions (vertically and horizontally) using a joystick. A vertical tracking task (VTT) in which an object needs to be tracked using a throttle device in one dimension (horizontally). The session also includes a multitasking assessment requiring applicants to complete the DLT and two tracking tasks simultaneously. The final multitasking assessment requires the applicants to respond to emergency scenarios with specific inputs on the stick and throttle while simultaneously completing the two tracking task. The Skill Factor score is a combination of the tasks in this session. A detailed description of these tests is outside the scope of this paper, and they are included only to provide context for evidence of incremental validity of the DOT beyond these measures. The relationship between the different components of the PBM and the performance of student Naval Aviators during primary training were examined. The data include 4,826 student Naval Aviators who began primary; 1,511 completed a previous version of the ASTB but were given the PBM as part of the initial validation of the current test. For these individuals, the PBM was given using the same equipment and computers used to administer the new version of the ASTB. Unlike the other 3,315 student Naval Aviators, their PBM scores were not included as selection criteria. They were already selected for aviation training based on scores from a previous version of the ASTB, which did not include the PBM.

Analyses were limited to student primary completion status and primary phase NSS. It is also important to note that the ASTB scores used for all the applicants were only the initial ASTB attempts. Approximately 20% of applicants take the battery multiple times (up to three attempts) to try and improve their scores with the most recent exam scores used to select candidates.

Table 1 presents the means, standard deviations and inter-correlations for the two criterion variables, completion (dichotomous) and NSS, as well as the five factors that are used to measure performance within the PBM. All the subtests within the PBM are significantly correlated with both completion and NSS. Table 2 shows the change in DOT Factor for both all applicants and students who began primary for each year we have data on. A hierarchical regression was performed to evaluate how much incremental validity DOT adds to the other four component scores of the PBM when predicting NSS. The first stage of the analysis, the PBM without DOT significantly contributed to the regression model, F(4,4358) = 126.891, *p* < .001 and accounted for 10.4% of the variance in NSS. The addition of the DOT to the model increased the variance accounted for by 1.9% and this change in *R*^2^ was significant, *F*(5,4357) = 122.913, *p* < .001. A hierarchical regression was then performed comparing all the PBM tests with and without DOT across the year the exam was taken, to look at whether DOT’s incremental validity is changing over time. Due to the length of time between application and completion of primary school, as well as a delay in obtaining primary school data, the majority of the applicants took the ASTB-E or were part of the PBM validation study prior to 2017. This analysis showed that DOT added significant incremental validity to the PBM in 2013, 2014, and 2015, however failed to add incremental validity in 2016 and 2017. The full results of the hierarchical regression for year are presented in Table 3. The Skill Factor which did not show a significant contribution in any of the years was included in the PBM model since it is currently used as part Navy aviator selection.

**Table 1.** Descriptive statistics and intercorrelations among subtests of the PBM and outcome variables for primary school

VarIable	M	SD	1	2	3	4	5	6	7
1. Completion	0.91	0.28	-						
2. Primary NSS	49.58	9.85	0.08	-					
3. DOT Factor	158.93	37.86	0.10	0.23	-				
4. ATT Factor	36.08	19.49	0.07	0.29	0.22	-			
5. VTT Factor	48.56	14.80	0.06	0.22	0.19	0.56	-		
6. DLT Factor	33.48	9.35	0.06	0.16	0.26	0.17	0.17	-	
7. Skill Factor	15.79	9.70	0.06	0.15	0.22	0.26	0.26	0.27	-

Note: All correlations significant at the *p* < .01 level. Correlations with completed used full sample (N = 4826). All other correlations and means are with only students who finished primary (N = 4363)

**Table 2.** Annual breakdown of DOT Factor’s mean performance for all applicants and selected aviators, and primary training attrition and completion counts

Test Year	Applicant Mean (SD) DOT Factor Score	Selected Mean (SD) DOT Factor Score	Count Completed Training (Started Training)
2013	139.9 (45.7)	148.3 (39.9)	575 (658)
2014	139.9 (46.8)	155.1 (38.9)	1793 (1985)
2015	142.1 (47.2)	164.2 (35.1)	1118 (1211)
2016	143.9 (47.2)	167.0 (33.8)	743 (674)
2017	144.2 (48.4)	169.7 (37.0)	218 (192)
2018	149.6 (48.9)	-*	9 (9)
2019	152.6 (49.3)	-*	2 (2)
2020	156.0 (49.2)	-*	0 (0)

*Calculations were not possible for these elements due to the limited sample size.

**Table 3.** Hierarchical regression results for DOT incremental validity

		Model 1			Model 2		
		*B*	*SE B*	*β*	*B*	*SE B*	*β*
2013	ATT Factor	0.14	0.02	0.29**	0.14	0.02	0.27**
	DLT Factor	0.07	0.04	0.08	0.05	0.04	0.06
	VTT Factor	0.04	0.03	0.07	0.04	0.03	0.06
	Skill Factor	0.04	0.04	0.04	0.03	0.04	0.04
	DOT Factor				0.02	0.01	0.10*
	R^2^	0.13			0.14		
	F for change in R^2^	22.04**			6.17*		
2014	ATT Factor	0.12	0.01	0.25**	0.11	0.01	0.22**
	DLT Factor	0.08	0.02	0.08**	0.05	0.02	0.05*
	VTT Factor	0.04	0.02	0.07*	0.04	0.02	0.06*
	Skill Factor	0.05	0.02	0.05	0.03	0.02	0.03
	DOT Factor				0.04	0.01	0.16**
	R^2^	0.11			0.13		
	F for change in R^2^	54.94**			50.24**		
2015	ATT Factor	0.09	0.02	0.17**	0.08	0.02	0.16**
	DLT Factor	0.13	0.03	0.11**	0.10	0.03	0.08**
	VTT Factor	0.05	0.02	0.08*	0.05	0.02	0.07*
	Skill Factor	0.01	0.03	0.01	0.00	0.03	0.00
	DOT Factor				0.04	0.01	0.15**
	R^2^	0.07			0.09		
	F for change in R^2^	21.74**			26.75**		
2016	ATT Factor	0.12	0.02	0.24**	0.11	0.02	0.23**
	DLT Factor	0.10	0.05	0.08*	0.09	0.05	0.07
	VTT Factor	0.01	0.03	0.02	0.01	0.03	0.02
	Skill Factor	0.06	0.04	0.06	0.05	0.04	0.05
	DOT Factor				0.02	0.01	0.07
	R^2^	0.09			0.09		
	F for change in R^2^	15.77**			3.59		
2017	ATT Factor	0.09	0.04	0.16*	0.09	0.04	0.15*
	DLT Factor	0.23	0.08	0.20**	0.18	0.09	0.16*
	VTT Factor	0.12	0.06	0.16	0.11	0.06	0.15
	Skill Factor	0.16	0.09	0.13	0.12	0.10	0.10
	DOT Factor				0.03	0.03	0.10
	R^2^	0.21			0.21		
	F for change in R^2^	12.11**			1.51		

* *p* < .05, ** *p* < .01

## Study 2 DOT strategy study

### Method

#### Participants

A total of 133 US Sailors and Marines participated in the study, 14 of whom were female. Participants came from two different groups: 98 student Naval Aviators and 35 student air traffic controllers (ATC). The student Naval Aviators were all officers, with a mean age of 24.3 years (*SD* = 2.2 years), and included 10 females in the aviator group. The second group were enlisted ATC students with a mean age of 21.4 years (*SD = *4.3 years), four of whom were female. The study was approved by the institutional review board at the Naval Research Laboratory.

#### Procedure

The larger study consisted of a series of baseline eye tracking measurements and cognitive tasks that are outside the scope of this paper. The sequence of tasks will be described at a high level to provide some context for the overall experiment and data collection setting. Participants completed the task in a small computer lab and were tested in groups of 5–15 participants per session. Participants were always tested with their own cohort (i.e., ATC students only tested with other ATC students). After signing an informed consent form, participants were walked through how to set up and calibrate the eye tracking devices (Gazepoint GP3 HD). After the eye tracking devices were calibrated, baseline measurements for system accuracy and pupillary response to changes in luminance were collected. After the eye tracker calibration and baseline measurements, which took approximately 10 minutes, the participants completed the DOT. The DOT presented to participants was a slightly modified version of the one administered as part of the ASTB. The DOT version in the ASTB is web-based, whereas the version used for this study was designed to run on the local machine and integrate with the eye tracker. The task layout was the same, the instructions were the same, and size of all the images was the same. The number of practice and test questions was the same as the ASTB version, but the order of the questions was different from the ASTB DOT. The image from Figure 1 was taken from the version of DOT used for the current study. However, for this version of the DOT, there was a 4 second delay between DOT trial presentations when participants stared at the screen so that baseline eye tracking data could be collected. Pupillometry has been previously collected during the DOT to examine within task learning (Foroughi, Sibley, & Coyne, 2017), and the delay between trials was added to allow for a better separation of the tonic and phasic changes in pupil diameter during the task. A discussion of the physiological basis of changes in pupil size and its relation to cognitive effort are not relevant to this paper on spatial ability.

After going through the self-paced instruction slides, participants completed 12 practice trials in which they were provided feedback on their performance. Following the practice trials, the participants then completed 48 test trials in which no feedback was provided. The order of the practice and trial questions were the same for all participants. Once the participant completed all the DOT trials, they took a survey about the task. The initial three questions were intended for participants not pursuing a career in aviation. These three questions asked whether they had taken a military selection test, which included the DOT (all the aviators would have taken the ASTB). It also asked about prior aviation or navigation experience, and whether they were pursuing a career in aviation. The remainder of the questions, 4–7, was analyzed for this paper. Question 4 asked “What percentage of trials do you believe that you answered correctly?” Question 5 asked “Did you attempt to use a strategy to solve each problem in the Direction Orientation Task?” Question 6 asked “Did you switch strategies during the Direction Orientation Task?” Question 7 was multipart and asked “On what percentage of the trials did you apply the following strategies? (Please sum to 100)” There were 6 strategies to choose from: 1) Math (addition or subtraction), 2) Mental rotation (rotating one or both of the images), 3) Determined the cardinal direction of the parking lots immediately to your left or right then solved for the target parking lot, 4) Identified the North parking lot and then solved for the target parking lot, 5) Guessing, and 6) Other (with space to specify what the other strategy was).

Following the DOT, participants completed a modified version of Larson, Merritt, and Williams (1988) Mental Counters Task, and three attention control tasks: the anti-saccade, visual arrays, and sustained attention to control task (SACT) (Draheim, Tsukahara, Martin, Mashburn, & Engle, 2021). Results from these tasks are outside the scope of this paper and will not be discussed. The overall experiment took approximately 90 minutes; however, the DOT was the first task participants completed after the eye tracking system was set up, calibrated, and the baseline measures were gathered.

### Results

#### DOT performance

**Figure 4.**
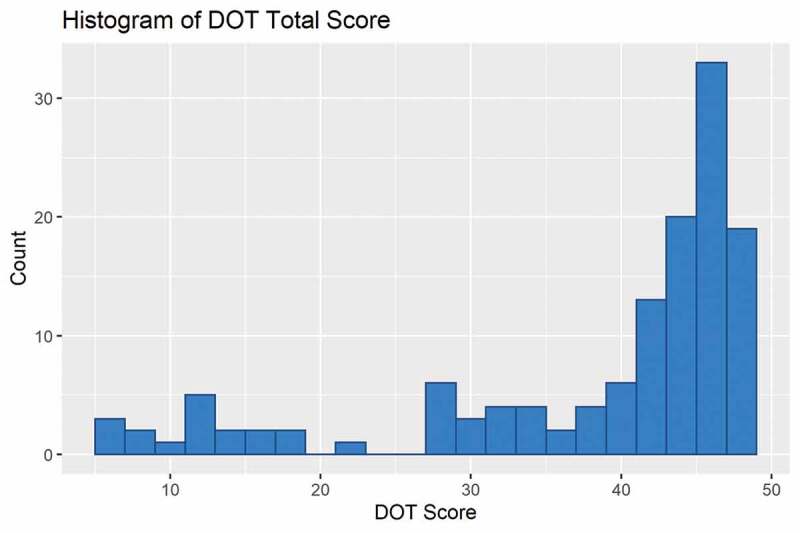
Histogram of DOT trial accuracy for all participants.

**Figure 5.**
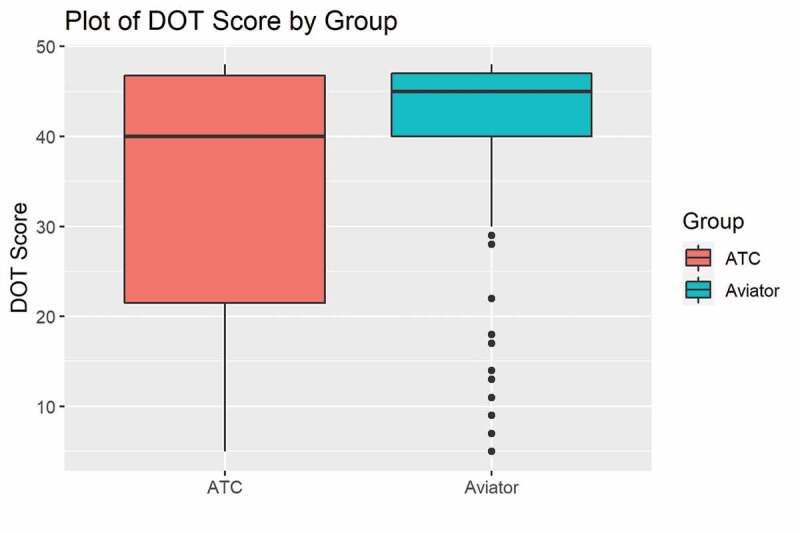
Box and whisker plot of DOT score by student type.

**Figure 6.**
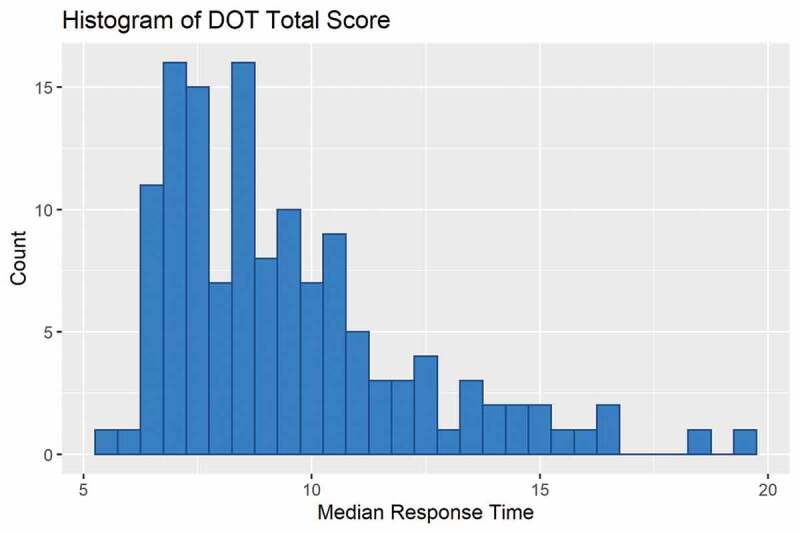
Histogram of median DOT response time for all participants.

**Figure 7.**
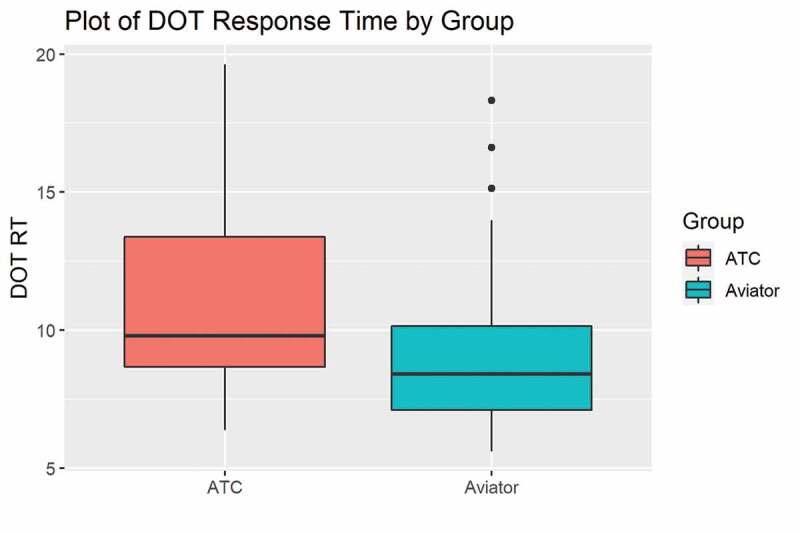
Box and whisker plot of DOT median response time (seconds) by student type.

DOT total was corrected on the 48 trials and the median response time was computed for all the participants. The average score was 38.79 (*SD* = 11.79). Figure 4 depicts the histogram of DOT scores for all participants. The mean score for the ATC students was 34.38 (*SD *= 14.59), and the mean for the student aviators was 40.32 (*SD = *10.30). Figure 5 depicts a box and whisker plot to show the distribution of scores across the two groups. The median response time for all DOT participants was 9.46 s (*SD = *2.71 s). Figure 6 shows a histogram of the average median response times for all participants. The average median response time for the ATC students was 11.1 s (*SD = *3.24 s), and the average median response time for the aviators was 8.9 s (*SD = *2.27 s). Given the unequal group size, a statistical comparison of the groups was not performed but is visualized in the box and whisker plot in Figure 7.

#### DOT survey

All the aviators reported previous exposure to the DOT and none of the ATC students reported previous exposure to the DOT. Both groups tended to underestimate their performance on DOT: the average estimated score for the aviators was 35.28 (*SD = *11.38), and the estimated score for ATC students was 30.01 (*SD* = 14.33). The average absolute difference between the actual score and estimated score for the student aviators was 7.47 (*SD = *7.74) and for ATC the average difference was 7.00 (*SD = *7.05). There was a significant correlation between estimated score on DOT and the actual score (*r = *.698, *p* < .001).

Question 7 asked for the percentage of time the different strategies were applied and asked participants to sum to 100. However, 26 participants entered values that did not sum to 100. All the responses were therefore scaled by taking the amount of time they claimed to spend, divided by the sum of all the values they entered.

Those strategies ranked from most frequently used to least used are: 1) identifying the cardinal direction(s) closest to the UAV’s heading (37.6%), 2) a rotation strategy (34.8%), 3) identifying the north parking lot and solving for the target parking lot (12.7%), 4) guessing (6.7%), 5) other (4.0%), and 6) math (3.4%). The use of the cardinal direction strategy was the only strategy positively correlated with performance on the DOT. The guessing strategy and the determining the North strategy were both negatively correlated with performance. Table 4 shows the average percentage of time that the different groups reported using each of the six different strategies. The amount of time spent on several of the reported strategies was significantly correlated with DOT performance. Table 5 shows the correlations of all the different strategies and DOT score and response time.

**Table 4.** Reported percentage of time spent using different strategies for each group

	Math		Rotation		Cardinal		North		Guess		Other	
	Mean	SD	Mean	SD	Mean	SD	Mean	SD	Mean	SD	Mean	SD
**Aviators**	2.9	4.8	32.7	29.7	42.3	33.3	11.5	18.8	6.3	15.2	4.3	18.8
**ATC**	4.9	10.3	40.8	24.6	24.4	20.8	15.9	17.5	7.7	11.9	3.4	11.7
**Combined**	3.4	6.7	34.8	28.6	37.6	31.5	12.7	18.5	6.7	14.4	4.0	17.2

**Table 5.** Correlation of DOT performance and DOT survey

	DOT Score	DOT RT	Math	Rotation	Guess	Cardinal	North	Other	Estimated Score Difference	EstimatedScore
**DOT Score**										
**DOT RT**	−.316**									
**Math**	.008	.201*								
**Rotation**	.056	.060	−.028							
**Guess**	−.261**	−.005	−.001	−.147						
**Cardinal**	.262**	−.143	−.154	−.570**	−.245**					
**North**	−.420**	.096	.019	−.234**	−.029	−.297**				
**Other**	.132	−.136	−.059	−.190*	−.097	−.241**	−.098			
**Estimated Score Difference**	−.336**	−.072	−.085	−.030	.011	.009	.167	−.004		
**Estimated Score**	.698**	−.356**	−.057	.031	−.240**	.257**	−.274**	.123	.440**	

**Correlation is significant at the 0.01 level (2-tailed).

*Correlation is significant at the 0.05 level (2-tailed).

## Discussion

The purpose of this paper was to evaluate the DOT, specifically looking at two key issues: 1) the effectiveness of the test over time, and 2) whether the use of non-spatial strategies was positively correlated with performance. The distribution of scores was examined using historical data from Navy, Marine Corps, and Coast Guard applicants on the DOT. The relationship between the use of non-spatial strategies and DOT performance was examined by collecting experimental data on the use of different strategies and correlating percent of time spent on those strategies with DOT performance.

Although previous papers discussed a ceiling effect being one of the limitations of the DOT (e.g., Keiser et al., 2019), the only empirical evidence was from performance on the DOT by a group of student aviators. The data presented in this paper illustrate a clear ceiling effect on the DOT for applicants as well. The applicant data also showed a significant improvement in DOT performance since the test was first implemented. Scores have increased from a mean of 67.8% correct across applicants taking the test in 2014 (the first full year of DOT for the Navy) to a mean of 75.6% correct across applicants taking the test in the first nine months of 2020. Across the first five years of applicant data, 11% of all applicants made two mistakes or fewer on the DOT (over 95% correct), in the nine months of data for 2020, 22% of the over 3,000 applicants for 2020 met or surpassed this 95% benchmark.

Applicants are not asked specifically if they have ever seen or practiced the DOT prior to taking the ASTB; however, this performance data suggests that the applicants may be changing their approach to DOT since the test was first implemented and have possibly been given or devised their own strategies for quick, correct responses during the test. Further, the analysis of the available criterion data shows the DOT no longer contributing incremental validity to the PBM’s prediction of NSS after 2015. However, given the small sample size for 2017 it is unclear if these findings on DOT’s lack of incremental validity will continue, once more data beyond 2017 is made available. Taken together, the applicant data and the available validity data suggest that there is a potential problem with the test.

The second study in this paper examined how performance was related to reported strategy use. The only strategy positively correlated with performance on the DOT was the cardinal direction strategy. The cardinal direction strategy was specifically phrased as “Determined the cardinal direction (e.g., N, E, S, W) of the parking lots that were immediately to your left and/or right and then solved for the target parking lot.” If the UAV is flying a cardinal direction, then the parking lot at the top of the camera feed is that direction. If the UAV is flying a non-cardinal direction, e.g., 120 degrees, the cardinal directions that the heading lies between (90 and 180 degrees) are the two parking lots on the top half of the UAV screen. The one to the right is the one with an orientation greater than the heading (South/180), and the one to the left is the one with an orientation smaller in value than the heading (East/90). The cardinal direction strategy is more of an analytic strategy than a spatial one. A variation on the cardinal direction strategy is to count from the UAV heading where the target parking lot should appearfor example, counting the cardinal directions that appear clockwise from the UAV’s heading on the map and then doing the same on the camera feed.

Using non-spatial strategies on spatial tasks is a common issue associated with the assessment of spatial abilities (Hegarty, 2010). However, as the only measure of spatial ability for Navy and Marine Corps aviator applicants, there is added importance that the DOT actually assesses spatial ability. The ASTB contains multiple tests of cognitive ability and the DOT is meant to specifically capture spatial ability. If applicants are using different strategies it could impact the construct validity of the DOT, since the DOT could be measuring different constructs for different individuals. Neither study in this paper directly addressed whether DOT is measuring spatial ability or whether the use of different strategies impacts the DOT’s construct validity. Future research still needs to address these questions.

The applicant data presented here suggest that applicants have been shifting their approach to this test over time resulting in significant improvements in their DOT scores. Further research needs to be done to determine if the increasing use of strategies such as the cardinal direction strategy may be accounting for the change in DOT applicant scores.

One of the intended advantages to using cognitive ability tests such as the DOT, is that unlike tests of crystallized intelligence, they have relatively smaller subgroup differences (Barron & Rose, 2013). If either strategy, preparation, or advanced knowledge of the test are contributing to the diminished effectiveness of the test, it may lead to an unfair advantage for applicants who have greater access to information about the ASTB. While there is no direct evidence of changes in subgroup difference scores on the DOT, it is a concern if advance knowledge of the DOT is what is contributing to its diminished effectiveness.

Having test applicants aware of the test in advance creates a number of challenges specific to the DOT. An alternative explanation of the change in test scores over time is that participants are using a piece of scrap paper to draw a compass rose and then rotating it based upon the vehicle heading. A YouTube video showing this strategy has more views than the total number of individuals who have taken the ASTB (Davis, 2016). Participants are allowed to use paper in sections of the ASTB taken before the DOT and are not explicitly told they cannot use scrap paper during the DOT. This strategy effectively removes any mental rotation from the task.

One of the limitations of the empirical study reported here is the lack of additional tests of spatial ability to directly evaluate the construct validity of the DOT. One of the questions raised by the results from the experimental study on strategy use is whether training individuals on a strategy such as the cardinal direction strategy can impact their performance and reduce the test’s correlation with other spatial ability tests. Future research should examine whether participants trained with different instructions/strategies can outperform those trained with the standard instructions. The presence of practice versions of the test online negates the importance of using standardized instructions; the instructions would only matter if everyone’s first official attempt at the DOT was their first time seeing its content and instructions.

This study shows that performance on the DOT is correlated with Navy Training performance in primary school and that the DOT adds incremental validity over the other portions of the PBM. However, it also shows evidence that this incremental validity is no longer present in applicants after 2015. Other researchers have also shown that the DOT adds incremental validity when predicting aviation training success (Barron & Rose, 2013; Carretta, 2005). However, this paper highlights several concerns associated with the DOT that call into question its continued use. The rising scores on the test suggest that it is becoming less effective at differentiating applicants based on spatial ability. Although there have been two attempts to update the DOT and increase its difficulty by Keiser et al. (2019) and Coyne et al. (2020), these updated versions suffer from the same potential strategy issues as the version of the DOT currently in use for selection. If spatial ability is indeed an important construct for aviators, then further testing should be pursued to investigate whether the DOT is adequately assessing the construct, or whether new alternative tests need to be developed. The military must assume that any applicant taking the ASTB is motivated and will have practiced any of the tests that he or she can. Having a test like the DOT that is vulnerable to practice effects, non-spatial strategies, and simple visual aids appears to have limited the usefulness of the test. The paper raises some serious concerns with the DOT and presents evidence that the test may have become compromised over time.

## Data Availability

Operational selection and classification data are not available for public release without written consent from the Department of the Navy.
